# Determinants of Under-Five Child Mortality in Ethiopia: Analysis Using Ethiopian Demographic Health Survey, 2016

**DOI:** 10.1155/2020/7471545

**Published:** 2020-09-18

**Authors:** Addisalem Tebeje Zewudie, Abebaw Addis Gelagay, Engidaw Fentahun Enyew

**Affiliations:** ^1^Department of Population Studies, College of Social Sciences and Humanities, University of Gondar, Gondar, Ethiopia; ^2^Department of Reproductive Health, Institute Public Health, College of Medicine and Health Sciences, University of Gondar, Gondar, Ethiopia; ^3^Department of Human Anatomy, School of Medicine, College of Medicine and Health Sciences, University of Gondar, Gondar, Ethiopia

## Abstract

**Background:**

Under-five mortality rate is a leading indicator of the level of child health and the overall development in countries which indicate the quality of life of a given population, as measured by life expectancy.

**Objectives:**

To identify and analyze factors that may have a significant influence on under-five mortality in Ethiopia.

**Methods:**

A national representative cross-sectional study and a quantitative study were conducted among 18,008 households selected based on 2016 EDHS data. The analysis was done using SPSS version 20 statistical software. Both bivariate and multivariable analyses were employed. In multivariable analysis, *p* value less than 0.05 was considered statistically significant and odds ratio with 95% CI (confidence interval) was used to assess the determinants of under-five child mortality.

**Results:**

A total of 10,641 children were included in the study with a 99.0% response rate. The U5CM for being a rural resident (AOR = 1.802, 95% CI: 1.251, 2.595), not breastfeeding (AOR = 2.956, 95% CI: 2.490, 3.511), having multiple birth (AOR = 4.755, 95% CI: 3.440, 6.572), male gender (AOR = 1.363, 95% CI: 1.153, 1.612), having first birth order (AOR = 1.592, 95% CI: 1.275, 1.992), and having family size six and above (AOR = 2.187, 95% CI: 1.769, 2.707). The increment of family size increases the risk of U5CM.

**Conclusion:**

Multivariate logistic analysis reflected that place of residence, mothers' educational level, religion, current breastfeeding status, type of birth, sex of child, birth order, and family size were found to be significant predictors of under-five child mortality. So, government policy, nongovernmental organizations, and all concerned bodies should be focused on the major determinants of under-five child mortality and put in a lot more effort to reduce under-five child mortality, and health intervention policies should be revised.

## 1. Introduction

### 1.1. Background

The main issues of the fourth and fifth Millennium Development Goal (MDG 4 and 5) are to reduce the under-five mortality rate and improve maternal health which by implication increase the chance of child survival [1]. More recently, the Growth and Transformation Plan of Ethiopia (GTPE) has outlined the strategic measures that can significantly contribute to reducing under-five mortality [1]. The GTPE MOH has started to make plans for reducing under-five mortality rates to below 30 deaths per thousand live births by 2035 [2]. Reaching this low level will require improvements in social-economic status of the population as well as improvement in the direct services provided by the health sector [3]. And the sustainable development goal (SDG) is aiming for an under-five mortality rate of 25 per 1000 living births or less by 2030. The under-five mortality rate is the probability (expressed as a rate per 1000 live births) of a child born in a specified year dying before reaching the age of five [4]. The first five years of life are the most crucial to the physical and intellectual development of children [5].

The under-five mortality rate is a leading indicator of the level of child health and the overall development in countries which indicate the quality of life of a given population, as measured by life expectancy, or understanding the determinant factors of under-five mortality is essential to inform public health policies and design strategies to accelerate the reduction of under-five mortality. This is particularly important because less than five mortality has long been used as one of the measures of the level of socioeconomic development of a nation (CSA and ICF, 2017) [6, 7].

The under-five mortality rate (U5MR) is generally 29 times higher in developing nations compared to developed countries [4]. Approximately 6.3 million infants and children under five years of age die each year, with large variations in under-five mortality rates and trends across regions and countries [7]. A 2013 report on child mortality states that about three-quarters of all child deaths happened in two regions Africa (46%) and Southeast Asia (28%). More than 50% of these deaths were clustered in only six countries: China, Democratic Republic of the Congo, Ethiopia, India, Nigeria, and Pakistan. On average, 1 out of every 11 children born in sub-Saharan Africa dies before age five. This is nearly 15 times the average rate (1 in 159) in high-income countries. The highest rates of under-five mortality are concentrated in sub-Saharan Africa and South Asia [8, 9].

Globally, under-five mortality has dropped significantly by almost 45 percent between 2009 and 2011, but this progress is not the reality for all countries, despite much progress in advanced countries. In recent years, however, under-five mortality has declined and even reversed in many sub-Saharan African countries while they have continued to improve in other regions [10]. The under-five-year-old mortality rate is going down all over the world. But there are about 11 million children under five years dead every year in developing countries. Two-thirds of deaths are preventable. Unsafe water, malnutrition, and lack of education, health care, and social service are the major factors [4].

The influencing factors of under-five child mortality are mainly socioeconomic, demographic, and biological factors. Socioeconomic factors mainly result in the erogeneity of child deaths, for example, uvula cutting, milk teeth extraction, female genital mutilation, and eyebrow incision after birth which is always closely related to socioeconomy and culture. Demographic factors affect both endogenous and exogenous deaths, like problems at birth which are difficult to prevent and control and exogenous deaths which can be prevented by public health measures, immunization, and antibiotic treatment. Biological factors mainly refer to the mother's fertility information and the total number of children born which are concerned at the family level rather than children [11, 12].

The mortality rate of children under five in Ethiopia has been declining. The decline of the role of agriculture in the national economy, the increase of urbanization, and the launching of globalization have accelerated the economic performance of the country and significantly changed the trend of mortality rates particularly the mortality rate of children under five [9].

The trend in demographic and health indicators from 2000 to 2016 EDHS shows improvement of child health status. Early childhood mortality (all neonatal mortality, postnatal mortality, infant mortality, child mortality, and under-five mortality) has been decreasing in Ethiopia. For example, under-five mortality has decreased from 123 deaths per 1000 live births in 2005 to 88 deaths per 1000 live births in 2011 and 67 percent deaths per 1000 live births in 2016 [8]. But even if mortality has decreased, it is not as fast as needed and insufficient to reach GTP and SDG. Still, Ethiopia is at a lower position than other East African countries in terms of many child health indicators [11].

As mentioned above, the under-five child mortality rate in Ethiopia is still very high. Despite numerous interventions and action plans, very little evidence exists on why infant and under-five child mortality rates in Ethiopia have not declined as desired [13]. If Ethiopia is committed to achieving the GTP and SDG on child mortality, it is necessary to understand clearly the factors that are contributing to the high levels of mortality.

Therefore, this study is aimed at addressing determinants of the major risk factors of under-five mortality taking into consideration various maternal, environmental, and socioeconomic characteristics and their effect on child mortality in Ethiopia, based on the 2016 Ethiopia Demographic and Health Survey data.

## 2. Objectives

### 2.1. General Objective

The general objective is to identify and analyze socioeconomic, demographic, and environmental factors that may have a significant influence on under-five mortality in Ethiopia.

#### 2.1.1. Specific Objectives

The specific objectives are to determine the major factors of under-five child mortality and to assess the magnitude of under-five child mortality.

## 3. Materials and Methods

### 3.1. Study Design

The study design was conducted based on a nationally representative cross-sectional study, and a quantitative study was employed. The sampling frame used for the 2016 EDHS was the Ethiopia Population and Housing Census, which was conducted in 2007 by the Ethiopia Central Statistical Agency (CSA). The sample for the 2016 EDHS was designed to provide estimates of key indicators for the country as a whole, for urban and rural areas separately, and for each of the nine regions and the two administrative cities.

The 2016 EDHS sample has selected a total of 18,008 households for the sample of which 17,067 were occupied; among the sample, 16,583 eligible women and 14,795 eligible men were identified in the sample household. The sample was stratified and selected in two stages. In the first stage, a total of 645 EAs (202 EAs in urban areas and 443 EAs in rural areas) were selected with probability proportional to the EA size (based on the 2007 PHC) and with independent selection in each sampling stratum.

In the second stage of selection, a fixed number of 28 households per cluster were selected with an equal probability systematic selection from the newly created household listing. All women age 15-49 and all men age 15-59, who were either permanent residents of the selected households or visitors who stayed in the household the night before the survey, were eligible to be interviewed. In all the selected households, height and weight measurements were collected from children (0-59 months), women (15-49), and men (15-59).

### 3.2. Source of Data

Secondary data was used for this study from the 2016 Ethiopia Demographic and Health Survey (EDHS) conducted in Ethiopia as part of the worldwide Demographic and Health Survey project. The survey collected demographic and health information from a nationally representative sample of women in the reproductive age group 15-49 and men aged from 15 to 59. The 2016 EDHS was the fourth demographic and health survey conducted in Ethiopia.

### 3.3. Study Area

The study was conducted in Ethiopia. Administratively, Ethiopia is divided into nine geographical regions and two administrative cities.

### 3.4. Variables in the Study

#### 3.4.1. Response Variable

The dependent (response) or outcome variable for this study is the survival time of under-five mortality measured in months from birth until death/censor (before age five). The response is binary: yes or no. As mentioned above, the dependent variables are dichotomous, coded as zero for censored observations (for those who died after five years old or those who are alive at the time of the survey) and coded as 1 indicating uncensored observation (for those who died before five years old) (alive = 0 and dead = 1).

#### 3.4.2. Explanatory Variables

The explanatory variables are used as predictors of under-five child mortality. Broadly, the researcher grouped the variables into four: socioeconomic, biodemographic, health-seeking behaviors, and environmental health determinants, which contribute to under-five child mortality.

#### 3.4.3. Methods of Data Analysis

The data was analyzed using SPSS version 20 statistical software. Descriptive analysis was made to describe the characteristics of the study participants. A binary logistic regression model was employed to analyze the determinants of under-five mortality in Ethiopia. Bivariate analysis was first done to the crude association between independent and dependent variables. Covariates having *p* value less than 0.2 in bivariate analysis were further entered into a multivariable logistic regression model. Multivariable analysis was done using backward stepwise logistic regression. *p* value less than 0.05 was used as statistically significant, and the odds ratio with 95% CI was used to assess the presence and strength of association between covariates and dependent variable.

## 4. Results

The initial population consisting of 10,752 children's information was obtained by interviewing face to face their mothers. A total of 10,641 children were included in the study with a response rate of 99.0%. Among these 1.0% were found missing value and excluded from the study. Out of which, 10,641 children have complete measurements and were considered in this study and others were excluded due to incompleteness of data on the variables which are considered in the analysis.

There were 3133 (29.4%) children born to mothers who had a piped source of drinking water, 2442 (22.9%) with spring source of drinking water, and 5066 (47.6%) who used tube well water and other sources of drinking water. There were 5966 (56.1%) and 4675 (43.9%) children born to mothers with toilet facility and no toilet facility, respectively.

Of the total of 10,641 children included in the study, 10,363 (97.4%) were born in single birth and 278 (2.6%) were born in multiple births. Of the total live birth, 376 (59.2%) and 259 (40.8%) of child death have occurred for male and female, respectively. Analysis of the birth order showed that 136 (21.4%) children were born in the first birth order, 248 (39.1%) of children were of the 2^nd^-4^th^ birth order, and the rest 251 (39.5%) of children were born to birth order above four.

Of the total of 10,641 children included in the study, 3082 (29.1%) were born to mothers of Orthodox religion, 5442 (51.1%) were born to mothers of Muslim religion, 1662 (17.1) were born to mothers of Protestant religion, and 255 (2.4%) were born to mothers of other religions. The result of the current contraceptive method showed that 2753 (25.9%) of children were born to mothers currently using contraceptive methods, and the remaining 7888 (74.1%) were children born to mothers not currently using contraceptive methods.

Of the total number analyzed, 7155 (67.2%) children were born at home, 3023 (28.4%) children were born at a public facility, and the remaining 463 (4.4%) children were born at private and other health facilities (Table 1).

143 (4.57%) children died to mothers who had a piped source of drinking water, 147 (6.02%) with a spring source of drinking water, and 345 (6.81%) who used tube well water and other sources of drinking water. 315 (5.28%) and 320 (6.84%) child deaths occurred in those with toilet facility and no toilet facility, respectively (Table 2).

484 (6.76%) children died from home delivery, 131 (4.33%) of death occurred from birth in public sectors, and 20 (4.32%) of child death occurred from birth in private sectors. Regarding the type of birth, from the total of 10,641 children included in the study, 10,363 (97.4%) were born in single birth and 278 (2.6%) were born in multiple. There were 58 (20.86%) and 577 (5.57%) child deaths occurring due to multiple and single births, respectively. With regard to sex of children, a higher U5CM was observed among male children (376 (6.86%)) compared to female children (259 (5.02%)) of the same age. Analysis of the birth order showed that 136 (21.4%) children were born in the first birth order, 248 (39.1%) of children were of 2^nd^-4^th^ birth orders, and the rest 251 (39.5%) of children were born to birth order above four.

From the total deaths, 136 (6.30%), 248 (5.32%), and 251 (6.58%) deaths were found from the birth orders 1, 2-4, and 5+, respectively. The child mortality rate is thus confirmed to be increasing steadily with birth order (Table 2). The increase in the child mortality rate with birth order may reflect a more intense competition faced by higher birth order of children in terms of caregiver time, medical resources, and nutritious food which are required by children.

Analysis of the family wealth index showed that 411 (6.80%) of child death occurred due to families having poor economic status, 87 (5.14%) of children died from families having medium economic status, and 137 (4.74%) of children died from families having rich economic status. The U5CM was found to be lower among breastfeeding children (393 (10.28%)) compared to nonbreastfeeding children (242 (3.55%)). Lower under-five mortality occurred in Orthodox (138 (4.48%)) and Protestant (101 (5.42%)) compared with Muslim (374 (6.90%)) and other religion followers (22 (8.63%)). As shown in Table 2, the family size, age of first birth, contraceptive method, and the death of under-five children are different in number. Higher U5CM were observed in family sizes 1-5 (7.45%) compared to family size above 6 (4.76%), and higher U5CM were observed in a mother's age of the first birth less than 20 (6.93%) compared to 20 years or above and noncontraceptive method-using households (6.53%) compared to households using a contraceptive method (Table 2).

Of the total of 10,641 children included in the study, 1974 (18.55%) were born in urban and 8667 (81.45%) were born in the rural part of Ethiopia. The higher percentage of death rate (568 (6.55%)) among children under five was recorded in the rural area, when compared to urban areas (67 (3.39%)) (Table 2).

### 4.1. Factors Associated with Determinants of U5CM

In bivariate analysis, place of residence, mother's educational level, source of drinking water, type of toilet facility, religion, wealth index, current contraceptive methods, and current breastfeeding status, type of birth, child sex, place of delivery, current marital status, birth order, family size, and region had *p* value less than 0.2. However, in multivariable analysis, place of residence, current breastfeeding status, type of birth, child sex, birth order, and family size had a statistically significant association with U5CM, while others failed to persist (Table 3).

The odds of under-five child mortality were two times (AOR = 1.802, 95% CI: 1.251-2.595) higher among rural residents as compared to the urban ones. The odds of under-five child mortality were three times higher (AOR = 2.956, 95% CI: 2.490-3.511) among noncurrently breastfeeding status as compared to currently breastfeeding status. The odds of U5CM were five times higher among children born with multiple births as compared to single birth (AOR = 4.755, 95% CI: 3.440-6.572). The odds of U5CM were two times higher among children born to male sex as compared to those children born with a female sex category (AOR = 1.363, 95% CI: 1.153-1.61).

The odds of U5CM were two times higher (AOR = 1.592, 95% CI: 1.272, 1.992) among children born with the first order as compared to those who were born from five and above birth orders. The odds of U5CM were two times higher among children born having family sizes six and above as compared to those children born from one to five family sizes (AOR = 2.187, 95% CI: 1.769-2.703).

## 5. Discussions

This study is aimed at identifying determinants of under-five mortality based on 2016 Ethiopian Demographic and Health Survey data. Both descriptive and binary logistic regression analyses were employed to examine factors affecting under-five mortality. From the study using a binary logistic regression model, we found that the factors that significantly affect under-five mortality in Ethiopia are place of residence, mother's educational level, source of drinking water, type of toilet facility, religion, wealth index, current contraceptive methods, current breastfeeding status, type of birth, child sex, place of delivery, current marital status, birth order, family size, and region.

The study shows that children living in rural areas face a higher risk of mortality than children living in urban areas. This is in line with the study based on the 2011 EDHS data, which used data from DHS for 24 African countries showing that most of the deaths of children occurred in rural areas [14–16]. The finding of the study showed that the odds of death of under-five children from children currently breastfeeding are significantly less than those of children not breastfeeding. The result was in line with the study done in Ethiopia [16, 17], a determinant of under-five child mortality based on 2011 EDHS data. Children born with multiple births had a higher risk of dying than those with single births, and it has a highly significant effect. A study in Ethiopia by Desta [10] showed that multiple births are highly correlated with high child mortality than single births. The risk of death of under-five female children was significantly different from that of male children. The mortality rate of boys appears to be more sensitive than the mortality rate of girls.

The study suggested that firstborn children experience a higher risk of dying than children whose birth order is five and above; children with birth order two up to four have a higher risk of dying than a child whose birth order is five and above in line with the study finding that birth order is one of the determinants of under-five child mortality showing that a firstborn child was exposed to a high risk of mortality [17, 18]. The study showed that children born in large family size are significantly associated with increasing the risk of under-five mortality relative to small family size. And this leads to the fact that as family size increases, the risk of under-five mortality also increases. This is because large households are more likely to share facilities. A similar study [18–20], “Determinant of Under-Five Child Mortality in Ethiopia” based on 2011 EDHS data, shows that as the family size increases, under-five mortality also increases.

## 6. Conclusions

Multivariate logistic analysis reflects that place of residence, mothers' educational level, religion, current breastfeeding status, type of birth, sex of child, birth order, and family size were found to be significant predictors of under-five child mortality. So government policy, nongovernmental organizations, and all concerned bodies should be focused on the major determinants of under-five child mortality and do a lot or more emphasis to reduce under-five child mortality, and health intervention policies should be revised.

## Figures and Tables

**Table 1:** Socioeconomic, biodemographic, environmental, and maternal health care variables determining U5CM in Ethiopia, 2016.

Variables	Category	Frequency	Percentage
Place of residence	Urban	1974	18.6
	Rural	8667	81.4
Mother's educational level	No education	6838	64.3
	Primary	2678	25.2
	Secondary	734	6.9
	Higher	391	3.7
Source of drinking water	Piped	3133	29.4
	Spring	2442	22.9
	Tube well water and others	5066	47.6
Availability of toilet facility	With facility	5966	56.1
	No facility	4675	43.9
Religion	Orthodox	3082	29
	Muslim	5442	51.1
	Protestant	1862	17.5
	Others	255	2.4
Wealth index	Poor	6059	56.9
	Middle	1693	15.9
	Rich	2889	27.2
Current contraceptive method	Yes	2753	25.9
	No	7888	74.1
Currently breastfeeding	Yes	3821	35.9
	No	6820	64.1
Type of birth	Multiple	278	2.6
	Single	10,363	97.4
Sex of child	Male	5483	51.5
	Female	5158	48.5
Place of delivery	Home	7155	67.2
	Public facility	3023	28.4
	Private and other facilities	463	4.4
Current marital status	Currently married	9903	93.1
	Currently not married	738	6.9
Mother's age at birth	15–19	404	3.8
	20–29	5332	50.1
	30–39	4057	38.1
	40–49	848	8
Birth order number	1	2167	20.4
	2–4	4661	43.8
	5+	3813	35.8
Number of visits	1	6338	59.6
	2–3	3591	33.7
	4+	712	6.7
Family size	1–5	4780	44.9
	6+	5861	55.1
Region	Tigray	1033	9.7
	Affar	1062	10
	Amhara	977	9.2
	Oromiya	1581	14.9
	Somali	1505	14.2
	Benishangul-Gumuz	879	8.3
	SNNP	1277	12
	Gambela	714	6.7
	Harari	605	5.7
	Addis Ababa	461	4.3
	Dare Dawa	547	5.1

**Table 2:** The distribution of child mortality by socioeconomic, biodemographic, environmental, and maternal health care variables in Ethiopia, 2016.

Covariates (explanatory variables)	Categories	Under-five child mortality (U5CM)			
		Live	Death	Total	% of U5CM status
Place of residence	Urban	1907	67	1974	3.39
	Rural	8099	568	8667	6.55
Mother's educational level	No education	6387	451	6838	6.6
	Primary	2538	140	2678	5.23
	Secondary	697	37	734	5.04
	Higher	384	7	391	1.80
Source of drinking water	Piped	2990	143	3130	4.57
	Spring	2295	147	2442	6.02
	Tube well water and others	4721	345	5066	6.81
Availability of toilet facility	With facility	5651	315	5966	5.28
	No facility	4355	320	4675	6.84
Religion	Orthodox	2944	138	3082	4.48
	Muslim	5068	374	5442	6.90
	Protestant	1761	101	1862	5.42
	Others	233	22	255	8.63
Wealth index	Poor	5648	411	6059	6.80
	Middle	1606	87	1693	5.14
	Rich	2752	137	2889	4.74
Current contraceptive method	Yes	2633	120	2753	4.36
	No	7373	515	7888	6.53
Currently breastfeeding	Yes	3428	393	3821	10.28
	No	6578	242	6820	3.55
Type of birth	Multiple	220	58	278	20.86
	Single	9786	577	10,363	5.57
Sex of child	Male	5107	376	5483	6.86
	Female	4899	259	5158	5.02
Place of delivery	Home	6671	484	7155	6.76
	Public facility	2892	131	3023	4.33
	Private and other facilities	443	20	463	4.32
Current marital status	Currently married	9321	582	9903	5.90
	Currently not married	685	53	738	7.18
Mother's age at birth	15–19	376	28	404	6.93
	20–29	5016	316	5332	5.93
	30–39	3822	235	4057	5.80
	40–49	792	56	848	6.60
Birth order number	1	2031	136	2167	6.30
	2–4	4413	248	4661	5.32
	5+	3562	251	3813	6.58
Number of visits	1	5943	395	6338	6.23
	2–3	3392	199	3591	5.54
	4+	671	41	712	5.76
Family size	1–5	4424	356	4780	7.45
	6+	5582	279	5861	4.76
Region	Tigray	992	41	1033	3.97
	Affar	972	90	1062	8.47
	Amhara	928	49	977	5.01
	Oromiya	1494	87	1581	5.50
	Somali	1402	103	1505	6.84
	Benishangul-Gumuz	815	64	879	7.28
	SNNP	1206	71	1277	5.56
	Gambela	670	44	714	6.16
	Harari	564	41	605	6.78
	Addis Ababa	447	14	461	3.04
	Dare Dawa	516	31	547	5.70

**Table 3:** Bivariate and multivariable analyses of variables associated with U5CM in Ethiopia based on EDHS, 2016.

Independent variables	Number of children		COR (95% CI)	AOR (95% CI)
	Alive	Death		
Place of residence				
Urban	1907	67	1.00	1.00
Rural	8099	568	1.996 (1.542-2.584)	1.802 (1.251-2.595)^∗^
Mother's educational level				
No education	6387	451	1.280 (1.053-1.556)	1.074 (0.858-1.344)
Primary	2538	140	1.130 (0.943-1.877)	0.956 (0.635-1.441)
Secondary	697	37	1.020 (1.823-8.230)	2.504 (1.105-5.674)
Higher	384	7	1.00	1.00
Source of drinking water				
Piped	2990	143	1.00	1.00
Spring	2295	147	1.528 (1.251-1.867)	1.172 (0.918-1.498)
Tube well water and others	4721	345	1.141 (0.935-1.393)	1.071 (0.859-1.335)
Availability of toilet facility				
With facility	5651	315	1.00	1.00
No facility	4355	320	1.318 (1.123-1.548)	0.935 (0.755-1.159)
Religion				
Orthodox	2944	138	1.00	1.00
Muslim	5068	374	2.014 (1.260-3.221)	1.727 (1.017-2.934)
Protestant	1761	101	1.279 (0.816-2.006)	1.459 (0.883-2.411)
Others	233	22	1.646 (1.018-2.663)	1.608 (0.968-2.671)
Wealth index				
Poor	5648	411	1.343 (1.059-1.704)	1.118 (0.852-1.467)
Middle	1606	87	1.462 (1.198-1.783)	1.211 (0.936-1.567)
Rich	2752	137	1.00	1.00
Current contraceptive method				
Yes	2633	120	1.00	1.00
No	7373	515	1.533 (1.250-1.879)	1.235 (0.980-1.558)
Currently breastfeeding				
Yes	3428	393	1.00	1.00
No	6578	242	3.116 (2.641-3.677)	2.956 (2.490-3.511)^∗^
Type of birth				
Multiple	220	58	4.471 (3.308-6.043)	4.755 (3.440-6.572)^∗^
Single	9786	577	1.00	1.00
Sex of child				
Male	5107	376	1.393 (1.183-1.639)	1.363 (1.153-1.612)^∗^
Female	4899	259	1.00	1.00
Place of delivery				
Home	6671	484	1.602 (1.314-1.952)	1.185 (0.934-1.503)
Public facility	2892	131	1.607 (1.017-2.539)	0.990 (0.597-1.640)
Private and other facilities	443	20	1.00	1.00
Current marital status				
Currently married	9321	582	1.239 (0.926-1.659)	1.114 (0.818-1.518)
Currently not married	685	53	1.00	1.00
Birth order number				
1	2031	136	1.052 (0.848-1.306)	1.281 (0.968-1.695)
2–4	4413	248	1.254 (1.046-1.503)	1.592 (1.272-1.992)^∗^
5+	3562	251	1.00	1.00
Family size				
1–5	4424	356	1.00	1.00
6+	5582	279	1.610 (1.370-1.892)	2.187 (1.769-2.703)^∗^
Region				
Tigray	992	41	1.00	1.00
Affar	972	90	1.454 (0.901-2.345)	1.583 (0.897-2.793)
Amhara	928	49	0.649 (0.426-0.989)	1.079 (0.686-1.698)
Oromiya	1494	87	1.138 (0.716-1.807)	1.237 (0.722-2.120)
Somali	1402	103	1.032 (0.677-1.573)	1.309 (0.825-2.077)
Benishangul-Gumuz	815	64	0.818 (0.541-1.237)	1.267 (0.812-1.978)
SNNP	1206	71	0.765 (0.491-1.191)	0.971 (0.592-1.594)
Gambela	670	44	1.020 (0.661-1.576)	1.182 (0.705-1.983)
Harari	564	41	0.915 (0.570-1.469)	1.208 (0.684-2.136)
Addis Ababa	447	14	0.826 (0.511-1.338)	0.953 (0.577-1.575)
Dare Dawa	516	31	1.918 (1.008-3.651)	1.018 (0.491-2.109)

Note: 1.00 = reference; ^∗^*p* values < 0.05.

## Data Availability

The datasets used and/or analyzed during the current study are available from the Ethiopian statistical agency and Ministry of Health.

## Abbreviations

CM: Child mortality
CSA: Central Statistical Agency
EA: Enumeration area
EDHS: Ethiopia Demographic Health Survey
EMDHS: Ethiopia Mini Demographic and Health Survey
FMOH: Federal Ministry of Health
GTPE: Growth Transformation Plan of Ethiopia
MDGs: Millennium Development Goals
MOFED: Ministry of Finance and Economic Development
MOH: Ministry of Health
NDHS: Nigeria Demographic Health Survey
NGOs: Nongovernmental organization
PHC: Population housing census
SDG: Sustainable development goal
U5MR: Under-five mortality rate
UN: United Nations
UNICEF: United Nations Children's Fund
USAID: United States Agency for International Development
WHO: World Health Organization.
